# Endoscopic treatment (endoscopic balloon dilation/self-expandable metal stent) vs surgical resection for the treatment of de novo stenosis in Crohn’s disease (ENDOCIR study): an open-label, multicentre, randomized trial

**DOI:** 10.1186/s13063-023-07447-1

**Published:** 2023-06-27

**Authors:** Carme Loras, Pablo Ruiz-Ramirez, Juan Romero, Xavier Andújar, Josep Bargallo, Esther Bernardos, Marta Maia Boscá-Watts, Carlo Brugiotti, Eduard Brunet, David Busquets, Elena Cerrillo, Francisco Javier Cortina, Juan Antonio Díaz-Milanés, Carmen Dueñas, Ramón Farrés, Thomas Golda, Ferran González-Huix, Joan B. Gornals, Jordi Guardiola, David Julià, Alba Lira, Jordina Llaó, Miriam Mañosa, Ingrid Marin, Mónica Millán, David Monfort, David Moro, Josep Mullerat, Mercè Navarro, Francisco Pérez Roldán, Eva Pijoan, Vicente Pons, José Reyes, María Rufas, Empar Sainz, Vicente Sanchiz, Anna Serracant, Eva Sese, Cristina Soto, Jose Troya, Natividad Zaragoza, Cristian Tebé, Marta Paraira, Emma Sudrià-Lopez, Vicenç Mayor, Fernando Fernández-Bañares, Maria Esteve, on behalf of the Grupo Español de Trabajo de la Enfermedad de Crohn y Colitis Ulcerosa GETECCU

**Affiliations:** 1 Endoscopy Unit, Department of Digestive Diseases, Hospital Universitari Mútua Terrassa, Plaça Dr Robert n° 5, Terrassa, Barcelona 08221 Spain; 2 Centro de Investigación Biomédica en Red de Enfermedades Hepáticas y Digestivas (CIBERehd), Madrid, Spain; 3 Consorci Sanitari de Terrassa, Terrassa, Spain; 4 Hospital General La Mancha Centro, Alcázar de San Juan, Ciudad Real Spain; 5 Hospital Clínico Universitario de Valencia, Valencia, Spain; 6 Hospital Comarcal d’Inca, Inca, Mallorca Spain; 7 Corporació Sanitària Universitària Parc Taulí, Sabadell, Spain; 8 Hospital Universitari de Girona Dr. Josep Trueta, Girona, Spain; 9 Hospital Universitari i Politècnic la Fe, Valencia, Spain; 10 Hospital Universitario de Cáceres, Cáceres, Spain; 11 Clínica de Girona, Girona, Spain; 12 Hospital Universitari de Bellvitge, Institut d’Investigació Biomèdica de Bellvitge (IDIBELL), L’Hospitalet de Llobregat, Spain; 13 Althaia, Xarxa Assistencial Universitaria de Manresa, Manresa, Spain; 14 Hospital Universitari Germans Trias i Pujol, Badalona, Spain; 15 Hospital de Sant Joan Despí Moisès Broggi, Sant Joan Despí, Spain; 16 IdISBa- Institut de Investigació Sanitaria de les Illes Balears, Palma, Spain; 17 Hospital Universitari Arnau de Vilanova, Lleida, Spain; 18 Unitat de Bioestadística, Institut d’Investigació Biomèdica de Bellvitge (IDIBELL), L’Hospitalet de Llobregat, Spain

**Keywords:** Crohn’s disease, De novo or primary stenosis, Endoscopic treatment, Balloon dilation, Self-expandable metal stent, Surgical resection, Randomized clinical trial, Proof-of-concept study

## Abstract

**Background:**

Stenosis is one of the most common complications in patients with Crohn’s disease (CD). Endoscopic balloon dilation (EBD) is the treatment of choice for a short stenosis adjacent to the anastomosis from previous surgery. Self-expandable metal stents (SEMS) may be a suitable treatment option for longer stenoses. To date, however, there is no scientific evidence as to whether endoscopic (EBD/SEMS) or surgical treatment is the best approach for de novo or primary stenoses that are less than 10 cm in length.

**Methods/design:**

Exploratory study as “proof-of-concept”, multicentre, open-label, randomized trial of the treatment of de novo stenosis in the CD; endoscopic treatment (EBD/SEMS) vs surgical resection (SR). The type of endoscopic treatment will initially be with EDB; if a therapeutic failure occurs, then a SEMS will be placed. We estimate 2 years of recruitment and 1 year of follow-up for the assessment of quality of life, costs, complications, and clinical recurrence. After the end of the study, patients will be followed up for 3 years to re-evaluate the variables over the long term. Forty patients with de novo stenosis in CD will be recruited from 15 hospitals in Spain and will be randomly assigned to the endoscopic or surgical treatment groups. The primary aim will be the evaluation of the patient quality of life at 1 year follow-up (% of patients with an increase of 30 points in the 32-item Inflammatory Bowel Disease Questionnaire (IBDQ-32). The secondary aim will be evaluation of the clinical recurrence rate, complications, and costs of both treatments at 1-year follow-up.

**Discussion:**

The ENDOCIR trial has been designed to determine whether an endoscopic or surgical approach is therapeutically superior in the treatment of de novo stenosis in CD.

**Trial registration:**

ClinicalTrials.gov NCT 04330846. Registered on 1 April 1 2020. https://clinicaltrials.gov/ct2/home

**Supplementary Information:**

The online version contains supplementary material available at 10.1186/s13063-023-07447-1.

## Background

Stenosis is one of the most frequent complications in patients with Crohn’s disease (CD), causing greater morbidity and increasing the likelihood of repeated surgery and short bowel syndrome [1–3]. Endoscopic balloon dilation (EBD) is the treatment of choice for a short stenosis located at the anastomosis from previous surgery [4–7]. However, there is no scientific evidence for determining the most appropriate treatment for de novo stenosis less than 10 cm in length (surgical versus endoscopic treatment), in terms of both efficacy and complications. Nor has it been established which of these two approaches has a greater impact on the quality of life of patients and on costs.

In many cases, a surgical approach allows for the removal of the entire inflamed intestine. However, the percentage of post-surgical recurrence 1 year after surgery is 80–85% [8], decreasing to 40% in patients who begin preventive immunosuppressive treatment immediately after surgery [9]. This means that more than 40% of patients will require combined immunosuppression to keep CD under control in the long term. In contrast, endoscopic treatment does not remove the affected intestine. Nevertheless, it has a prolonged therapeutic efficacy of 50–60%, with a very low percentage of complications (4–6%) [10].

A large number of studies have shown that the patient’s quality of life improves when CD is properly controlled, through either medical or surgical treatment [11]. However, there are no studies evaluating the quality of life of patients after endoscopic treatment. Nor are there comparative studies of the costs of the two procedures. However, a recent study comparing the cost of 38 endoscopic procedures with their surgical equivalent suggested that, in most cases, the cost of endoscopic treatment is four times lower [12]. The European Crohn’s and Colitis Organization (ECCO) guidelines on the management of stenosis in patients with CD consider that EBD and surgery are both suitable treatment options for terminal ileum short stenosis (< 5 cm), based on expert opinion (Level of Evidence 5), although there are no studies comparing the two forms of treatment [13].

A Spanish multicentre study coordinated by researchers involved in the current project, which includes one of the largest published series of EBD-treated CD patients, showed that therapeutic success with EBD in de novo stenosis was achieved in a large percentage of cases, similar to the results obtained with post-surgical stenosis (73% vs 84%) [14].

In addition, CD stenosis can be treated effectively with self-expandable metal stents (SEMS), and it has been suggested that these may be particularly indicated in patients who are refractory to balloon dilation, including both de novo and anastomotic stenosis patients [15–18]. Therefore, in order to compare the efficacy of these two endoscopic treatments, the ProtDilat study was carried [19]; it confirmed that both procedures are effective and safe for both post-surgical and de novo stenosis. Interestingly, EBD showed a significantly greater therapeutic superiority compared to fully covered SEMS (FCSEMS) when evaluating the results globally (80.5 vs 51.3%; primary end point). However, this difference was not observed in the sub-analysis of patients with stenosis > 3 cm (EBD: 66.7% vs FCSEMS: 63.6%). Moreover, EDB treatment had a significantly lower cost than FCSEMS (1365.63 euros vs 1923.55 euros, respectively). Therefore, SEMS may have a role to play in longer stenosis in which EBD has proven to be less effective.

This work was conceived as an exploratory proof-of-concept study, given that there are currently no studies comparing surgical and endoscopic approaches, making it difficult to calculate the adequate sample size. The aims of the study are to compare the impact on quality of life, complications, costs, and therapeutic efficacy of endoscopic (EBD/SEMS) vs surgical treatment for de novo stenosis in patients with CD.

## Methods/design

The ENDOCIR trial is a multicentre, open-label, randomized clinical trial with two parallel groups, without masking and with a 1:1 allocation ratio. Forty patients with de novo CD stenosis will be recruited from 15 hospitals in Spain and will be randomly assigned for endoscopic or surgical treatment. All Spanish tertiary and secondary centres with expertise in the management of inflammatory bowel disease were invited to participate through national meetings and e-mails. Finally, 15 of these centres agreed to participate in the study.

**Fig. 1:**
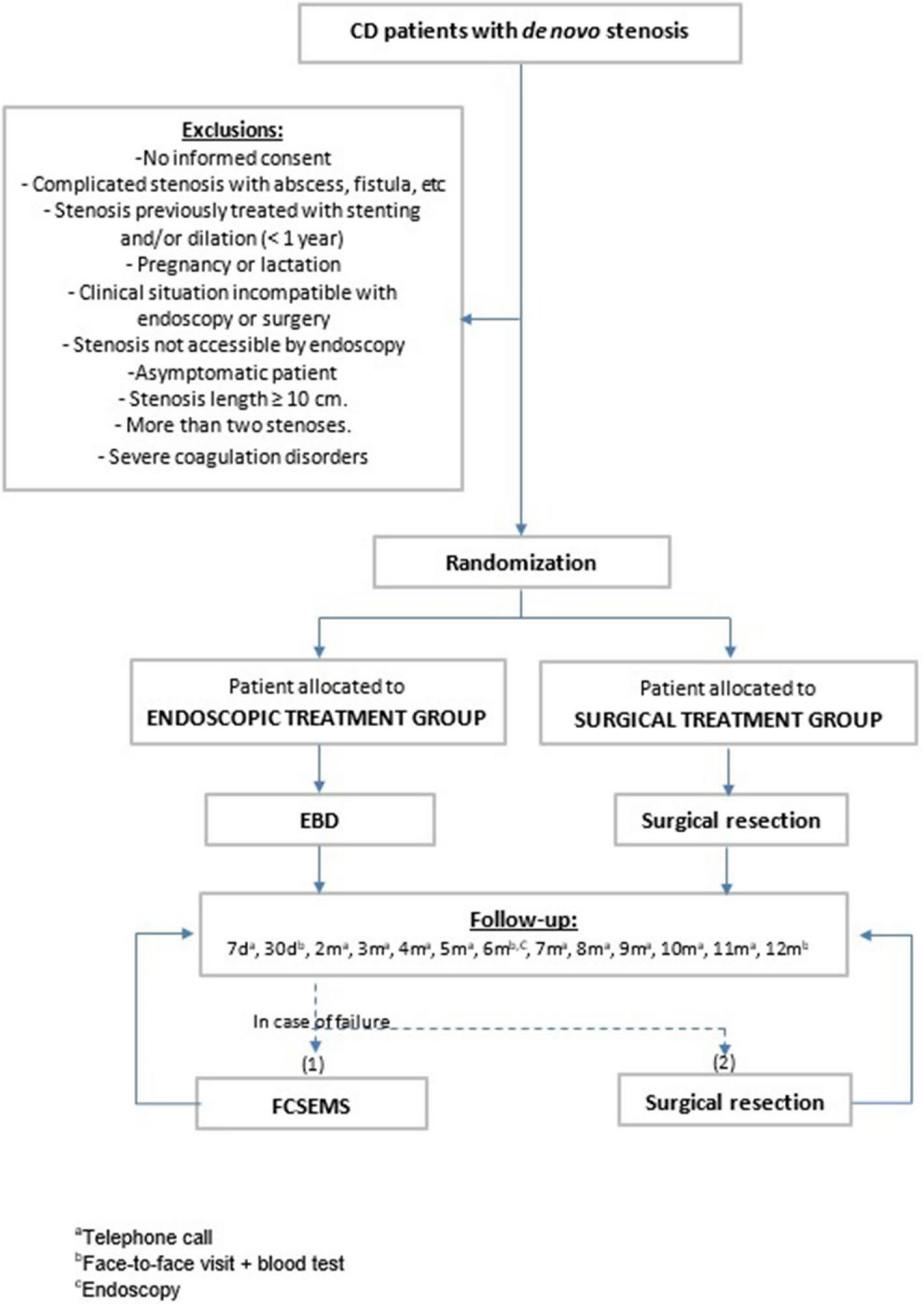
EndoCir flow chart

Central ethical approval of the study protocol has been confirmed by the Comitè Ètic d’Investigació Clínica (CEIC) of the Hospital Universitari Mútua Terrassa. The recruitment at other centres will not begin until local ethical approval has been obtained. A checklist with the recommendations for interventional trials (SPIRIT) is attached as an additional file (see Additional file 1). In Fig. 1, a flowchart of the study design is shown.

### Study population: patient identification, consent, and recruitment

To participate in this study, the patient has to be diagnosed with CD with de novo or primary (non-anastomotic) symptomatic stenosis, refractory to medical treatment.

The investigator at each centre will evaluate the inclusion of the patient in the study. The patient will be informed about the specifics of the study by knowledgeable personnel, who will help to resolve any questions that may arise. The informed consent form will be signed in the presence of participating personnel aware of all aspects of the study. The patient has the right to withdraw from the study at any time.

Principal investigators from each centre will have the task of presenting strategies to promote enrolment and to ensure that the study sample size is achieved.

The inclusion and exclusion criteria are listed in Table 1.

**Table 1:** Selection criteria

**Inclusion criteria:**
**Patients eligible for the trial must comply with all of the following at randomization**
- 18–80 years of age.
- Crohn’s disease with predominantly de novo fibrotic stenosis^a^ confirmed by endoscopic and radiological tests, accessible by endoscopy (colonoscope).
- Patients with known stenosis previously treated with stenting and/or dilation performed over 1 year before the date of inclusion.
- Symptomatology of intestinal occlusion-subocclusion.
- Refractoriness to conventional medical treatment (non-response to the standard accelerated step-up therapeutic approach).
- Stenosis length < 10 cm.
- Maximum of 2 stenoses.
- Informed consent from patient.
**Exclusion criteria**
- No informed consent from the patient.
- Complicated stenosis with abscess, fistula, or significant activity associated with CD not limited to the area of the stenosis.
- Patients with known stenosis previously treated with stenting and/or dilation performed < 1 year before the date of inclusion.
- Pregnancy or lactation.
- Any clinical situation that prevents the performance of endoscopy or surgery.
- Stenosis not accessible by endoscopy.
- Asymptomatic patient.
- Stenosis length ≥ 10 cm.
- > 2 stenosis.
- Severe coagulation disorders (platelets < 50,000; INR > 1.5).

^a^De novo stenoses are understood to be those stenoses not located in a surgical anastomosis. “Predominantly fibrotic stenoses” are understood to be those that do not present large ulcers at endoscopy (subscore SES-CD < 3) and that show minimal or no contrast uptake on MR enterography. In cases of stenosis with evident endoscopic and/or radiological activity, immunosuppressants and biologicals must have been previously used (see Inclusion criteria)

### Randomization and masking

Patients will be enrolled in this trial by gastroenterologists, surgeons, and endoscopists who will evaluate the cases in inpatient wards and in outpatient consultation areas. Randomization will be performed through an electronic database upon inclusion in the study. A code list will be generated by randomization with a 1:1 randomization ratio, by blocks. Each subject will be assigned a randomization code along with the treatment that corresponds to it. Once the patient meets the eligibility criteria and has provided informed consent, we will proceed to the allocation of each participant, in a centralized way, ensuring allocation concealment, and based on the randomization list. To prevent different subject recruitment rates at the various hospitals from interfering with the development of the study, the entire population will be randomized in blocks of four and two between the two treatment possibilities.

### Treatment and procedural technique

#### Pharmacological treatment

Patients in the endoscopic treatment arm will be maintained on the same pharmacological treatment that they were receiving at the time of the procedure whenever possible, adjusted for the occurrence of adverse effects (AEs) or loss of efficacy (e.g. occurrence of antibodies and undetectable drug levels). Patients in the surgery branch will receive treatment to prevent post-surgical recurrence in line with the recommendations of the Spanish Working Group on Crohn’s Disease and Ulcerative Colitis (GETECCU) [20]. They will also receive treatment with metronidazole 250 mg every 8 h for 3 months and with azathioprine at doses of 2–2.5 mg/kg/day. Patients intolerant to azathioprine will receive treatment with adalimumab at a rate of 40 mg every 2 weeks or infliximab at a rate of 5 mg kg every 2 months. If the stenosis is in the colon, biopsies will be performed to rule out malignancy.

#### General description of endoscopic technique

The type of endoscopic treatment will be EBD initially, and FCSEMS will be placed as a rescue endoscopic treatment if EBD failure occurs.

EBD treatment:

- Post-procedural admission in the short stay unit (SSU).
- Superficial sedation by endoscopist or anesthesiologist depending on the centre.
- Pneumatic balloon type: a CRE Boston scientific® (Marlborough, MA, USA) pneumatic balloon (CRE Wireguided Esophageal, Pyloric, Colonic, 8–20 mm; Cork, Ireland); diameter of the balloon at the endoscopist’s discretion.
- A maximum of 2 sessions of dilation will be performed with a minimum interval of 15–30 days between them.
- Dilation failure will be considered if more than 2 sessions of dilation are required.

FCSEMS treatment:

- Post-procedural admission to the SSU.
- Superficial sedation by endoscopist or anesthesiologist depending on the centre.
- FCSEMS type: a 22-diameter Taewoong fully covered, self-expandable metal stent (Niti-S S Enteral Colonic Stent, 6–10 cm; Gimpo-si, South Korea). The stent length will be set at the discretion of the endoscopist (at least 1.5 cm for each edge of the stenosis is necessary to ensure a successful procedure).
- To fix the stent. clips may be placed at the distal end of the stent at the endoscopist’s discretion.
- Maximum removal time of the stent will be 4 weeks, if spontaneous migration has not occurred.

#### General description of the surgical technique

- Procedure performed in line with the protocol of each centre.
- Whenever possible, laparoscopic resection will be the preferred surgical approach with Kono-S Anastomosis.

Surgical resection will also be performed as a rescue treatment in case of endoscopic treatment failure.

### Clinical evaluation and follow-up

#### Data collection and calendar

The collection of data for purposes of documentation will be carried out using a case report form (CRF), which will serve as an easily accessed source of information. After collection, the data will be introduced and managed using the REDCap electronic data capture tool hosted at Asociación Española de Gastroenterología (AEG; www.aegastro.es) [21, 22]. REDCap (Research Electronic Data Capture) is a secure, web-based software platform designed to support data capture for research studies, providing (1) an intuitive interface for validated data capture; (2) audit trails for tracking data manipulation and export procedures; (3) automated export procedures for seamless data downloads to common statistical packages; and (4) procedures for data integration and interoperability with external sources.

The collection of clinical information on the patients will begin at baseline and will continue with follow-up as established and defined in the study. Adverse events (AEs) will be noted from the beginning of the test until the conclusion of follow-up by means of scheduled controls. The following time-points and data items will constitute the data collection from the beginning through successive controls. The schedule of the study protocol is shown in Table 2.

**Table 2:** Data management, calendar

	**Screening and inclusion**	**Treatment**	**Follow-up**												
			**7d**	**30d**	**2 m**	**3 m**	**4 m**	**5 m**	**6 m**	**7 m**	**8 m**	**9 m**	**10 m**	**11 m**	**12 m**
Items															
Eligibility screen	X														
Informed consent	X														
Randomization	X														
Demographic data	X														
Clinical evaluation	X	X	X	X	X	X	X	X	X	X	X	X	X	X	X
Treatment procedure		X													
End-of-study evaluation															X
Type of appointment															
Face-to-face visit	X	X		X					X						X
Telephone			X		X	X	X	X		X	X	X	X	X	
Assessments															
Quality of life	X								X						X
AEs		X	X	X	X	X	X	X	X	X	X	X	X	X	X
Obstructive symptoms	X		X	X	X	X	X	X	X	X	X	X	X	X	X
Blood test	X			X					X						X
MRE	X														
Endoscopy	X								X						
Tobacco smoking	X		X	X	X	X	X	X	X	X	X	X	X	X	X
Medication	X	X	X	X	X	X	X	X	X	X	X	X	X	X	X

##### Time-points

Screening and Inclusion: Eligibly criteria will be assessed, and the number and extent of the stenoses together with the patient’s CD clinical status will be evaluated. In addition, the patients’ demographic data will be collected and quality of life pre-treatment assessed. Patients will be randomly assigned to a treatment group.

Treatment procedure: Relevant procedure-related information will be gathered, such as duration of procedure, materials, number and type of personnel involved, pharmacological treatment, and technical success. Pharmacological treatment, complications, and incidents will also be noted.

Follow-up: After treatment procedure, patients will be assessed in a face-to-face visit, or with a telephone call, on days 7 and 30, and every month until reaching 1-year follow-up. A record will be kept of the therapeutic requirements, complications, and/or incidents, as well as an assessment of active smoking status. In cases of tobacco addiction, an active policy aimed at elimination will be implemented.

A follow-up telephone call on the seventh day post-procedure and subsequently each month will be made to record AEs and the presence of obstructive symptoms. Face-to-face visits will be scheduled during the first and sixth months, and at the end of the follow-up (1 year). In addition to AEs and obstructive symptoms, a clinical assessment of disease activity (CDAI and inflammatory markers in blood) will be carried out. Moreover, an assessment of quality of life will be done at the sixth month and at the end of follow-up. Endoscopy will be performed 6 months after procedure to assess the occurrence of recurrence and escalation of treatment if necessary (in the case of surgical treatment: Rutgeerts index i2b-i4).

End of study: After completing 1-year follow-up or in case of discontinuation in the study, the following data will be compiled: reason for the end of follow-up, treatments performed, duration of follow-up, and treatment’s clinical success.

##### Data items

- Demographic data:
  The following data will be collected upon inclusion: sex, date of birth, age, and relevant medical history. For smokers, related information will also be collected (status, years of smoking, number of cigarettes per day).
- Quality of life:
  Patients’ quality of life will be evaluated with the 32-item Inflammatory Bowel Disease Questionnaire (IBDQ-32) and the EuroQol 5-Dimension 5-Level (EQ-5D-5L) questionnaires [23, 24]. Patient-reported outcome measures (PROMs) will be also collected using the IBD-Control questionnaire [25]. All the questionnaires may be found in Additional file 2.
- Activity of the disease:
  The clinical assessment of disease activity will be based upon biological markers in the blood (C-reactive protein (CRP), fibrinogen, and erythrocyte sedimentation rate (ESR), together with the CD activity index (CDAI). Faecal calprotectin will optionally be tested. Radiological assessment will be done by magnetic resonance enterography (MRE), and the Simplified Magnetic Resonance Index of Activity (MaRIAs) [26] will be calculated. All the MREs will be reread at the coordinating centre (Hospital Universitari Mútua Terrassa). The endoscopic activity of the disease will be quantified using the simple endoscopic score for Crohn’s disease (SES-CD) and Rutgeerts’ index, for non-operated and operated patients, respectively.
- Number, extent, and activity of the stenosis:
  Determination of the number and extent of the strictures will be made with colonoscopy and radiological techniques (MRE). Upon inclusion, the radiological activity at the site of stenosis will be calculated using the MaRIAs, and the endoscopic inflammatory activity of the area will also be described using the subscore SES-CD (non-ulcers, aphthous ulcers, large ulcers (0.5–2 cm), and very large ulcers (> 2 cm)). During follow-up, the following endoscopic variables will be collected: stenosis persistence, passage of the endoscope, and inflammatory activity of the area.
- Clinical recurrence:
  Assessment of clinical outcome will be made by applying the previously described Crohn’s Disease Obstructive Symptom Scale (Table 3) [15, 19].

**Table 3:** Obstructive Symptoms Scale

**Level**	**Description**
**0**	**No obstructive pain**
**1**	**Obstructive pain**^**a**^ No vomiting No complete bowel obstruction^b^ Occurring on less than 4 days during the last 8 weeks
**2**	**Obstructive pain**^**a**^ No vomiting No complete bowel obstruction^b^ Occurring on 4 or more days during the last 8 weeks
**3**	**Obstructive pain**^**a**^ Associated with vomiting Or complete bowel obstruction^b^ resolving Without hospitalization Occurring on 1 or 2 days during the last 8 weeks
**4**	**Obstructive pain**^**a**^ Associated with vomiting Or complete bowel obstruction^b^ resolving Without hospitalization Occurring on at least 3 days and fewer than 8 days During the last 8 weeks
**5**	**Obstructive pain**^**a**^ Associated with vomiting Or complete bowel obstruction^b^ resolving Without hospitalization Occurring on 8 days or more during the last 8 weeks
**6**	**At least one episode of complete bowel obstruction** Requiring hospitalization during the last 8 weeks

^a^Occurring after meals or increased by food, with intestinal noises that relieve the pain

^b^No passage of flatus associated with bloating

### Definitions

Technical success for endoscopic treatment is defined, for EBD and FCSEMS placement, as follows: for the former if the endoscope passes through the stricture after dilation, and for the latter the ability to place the stent on the stricture.

The success of the surgical technique is defined as the ability to perform the planned surgery.

AEs will be recorded in both the patient’s medical record and the electronic data collection tool using the appropriate medical terminology. Whenever possible, the diagnosis rather than the symptoms will be recorded. All AEs will be recorded once the informed consent has been signed and until 30 days after the last study visit. The study promoter/principal investigator will report all serious AEs within 7 days. In the event of a death, notification will be made within 24 h. Any instances of death during the follow-up will be investigated to rule out possible relation to the endoscopic or surgical treatment. Complications will be handled and treated in accordance with the decisions of the patient’s medical team. All additional tests and interventions will be duly documented.

The AEs for endoscopic treatment will be classified according to the AGREE classification [27]. (Table 4) and the American Society for Gastrointestinal Endoscopy (ASGE) lexicon [28] and will be reported per endoscopic procedure (EBD and/or FCSEMS). AEs will be considered related to the procedure when a causal relation is possible, probable, or definite, and they are to be graded as mild, moderate, or severe, as previously defined [28]. We will examine all patient outcomes during and following the endoscopic procedure for the development of post-procedure pain, broncho-aspiration, bradycardia, respiratory depression, cardiorespiratory arrest, arrhythmia, allergic reaction, bleeding, perforation, and death. Major bleeding related to the procedure will be considered if blood transfusion is required or if haemostasis is not achieved during the endoscopic procedure. Clavien-Dindo IIIa [29] will be used for the classification of surgery complications (Table 5).

**Table 4:** AGREE Classification for adverse events in GI endoscopy

**Grading**	**Definition**
No adverse event	• A telephone contact with the general practitioner, outpatient clinic, or endoscopy service without any intervention or • Extended observation of the patient after the procedure, <3 hours, without any intervention
Grade I	Adverse events with any deviation of the standard postprocedural course, without the need for pharmacologic treatment or endoscopic, radiologic, or surgical interventions. • Presentation at the emergency ward, without any intervention or • Hospital admission (<24 hours), without any intervention or • Allowed therapeutic regimens are drugs as antiemetics, antipyretics, analgesics, and electrolytes or • Allowed diagnostic tests: radiology and laboratory tests
Grade II	• Adverse events requiring pharmacologic treatment with drugs other than those allowed for grade I adverse events (ie, antibiotics, antithrombotics, etc) or • Blood or blood product transfusions or • Hospital admission for more than 24 hours
Grade III	Adverse events requiring endoscopic, radiologic, or surgical intervention
Grade IIIa	Endoscopic or radiologic intervention
Grade IIIb	Surgical intervention
Grade IV	Adverse events requiring intensive care unit/critical care unit admission
Grade IVa	Single-organ dysfunction (including dialysis)
Grade IVb	Multiorgan dysfunction
Grade V	Death of the patient

**Table 5:** Classification of surgery complications; Clavien-Dindo IIIa

**Level**	**Description**
**I**	Any deviation from the normal post-operative course not requiring surgical, endoscopic, or radiological intervention. This includes the need for certain drugs (e.g. antiemetics, antipyretics, analgesics, diuretics, and electrolytes), treatment with physiotherapy, and wound infections that are opened at the bedside
**II**	Complications requiring treatment with drugs other than those allowed for Grade I complications; this includes blood transfusion and total parenteral nutrition (TPN)
**III**	Complications requiring surgical, endoscopic, or radiological intervention Grade IIIa—intervention not under general anesthetic Grade IIIb—intervention under general anesthetic
**IV**	Life-threatening complications: this includes CNS complications (e.g. brain haemorrhage, ischaemic stroke, subarachnoid haemorrhage) which require intensive care, but excludes transient ischaemic attacks (TIAs) Grade IVa—single-organ dysfunction (including dialysis) Grade IVb—multi-organ dysfuncton
**V**	Death of the patient
**d**	If a patient continues to suffer from a complication at the time of discharge, the suffix “d” (for disability) is added to the respective grade of complication. This indicates that a full and careful follow-up is required to complete evaluation of the adverse event

### Outcomes

The primary outcome is to evaluate the patients’ quality of life at 1 year of follow-up. The secondary aim is to assess the rate of clinical recurrence, complications, and costs of both treatments at 1 year of follow-up.

Main variable: percentage of patients with an increase of more than 30 points in the IBDQ-32 quality of life index. Secondary variables: percentage of patients with clinical recurrence, percentage of complications, and costs.

### Sample size calculation

A sample size calculation has not been performed since the primary and secondary variable response rates are unknown, and this is therefore an exploratory “proof-of-concept” study. Based on the experience acquired by the research group in the ProtDilat study [19], and taking into account the participating centres, the number of patients to be included during the 2 years of recruitment is expected to be 20 in each arm, for a total of 40 patients.

### Statistical analysis

The demographic and clinical patient profiles will be summarized by the study group using descriptive statistics to assess the baseline comparability. Special attention will be paid to symptoms, disease activity, and location and length of the strictures.

Subjects will be deemed to have success in the primary outcome when an increase of 30 points or more on the IBDQ-32 quality of life index is observed at 1-year follow-up. The primary outcome will be compared by the study group in the intention-to-treat population and the per-protocol population using a *χ*^2^ test, and the relative risk (RR) and its 95% CI will be calculated. As a secondary goal, a generalized linear model will be used to assess whether there are variables that influence the quality-of-life assessment at 1-year follow-up. Moreover, the Kaplan-Meier analysis and a Cox regression model will be used to analyse differences between groups regarding time to clinical and endoscopic recurrence. The secondary outcomes (clinical recurrence and complications) will be analysed in the same way as the primary outcome.

Finally, the principal analysis will be repeated in the following subgroups of clinical interest with an exploratory and sensitivity purpose: sex, age group, location of the disease, and treatments undergone. *P* values of less than 0.05 will be deemed statistically significant. Data management and statistical analysis will be performed using R software version 4.1 or superior.

### Cost analysis

Information will be gathered and reported on the costs incurred by patients in each arm along the treatment pathway that they follow from randomization to the end of the trial follow-up (1 year). These pathway costs will include appointments at the hospital (inpatient and outpatient), plus any primary or community care appointments, medications, and emergency attention, and any other things that might be of interest to the intended decision-maker, as well as the costs of the initial interventions themselves. The cost of the initial interventions will be determined using the previously described direct cost calculation method [12]. This involves identifying the theoretical cost of the treatment received by the patient directly from the endoscopic office or surgical room and excludes indirect costs of the endoscopic or surgical unit itself, as well as the structural costs of the hospital. We will prospectively record the cost of all the resources required for the endoscopic and surgical procedures, immediate complications, and recovery period. Results will be reported as mean cost for both treatments.

### Other considerations

#### Withdrawal

AE or other clinical condition of the patient which, at the clinician’s discretion, warrants withdrawal of the patient from the study, pregnancy, or expressed wishes of the patient. Withdrawal from treatment will not mean suspension of the study, given that follow-up will be maintained until the end of the study in accordance with the protocol.

### Ethical aspects and confidentiality

The protocol will be approved by the ethical committee of each participating hospital as well as that of the coordinating centre. The study researchers will carry out their tasks in compliance with ethical principles of clinical research established in the Declaration of Helsinki, and with the rules of Good Clinical Practices. It is planned to hire a policy to cover the concepts and compensations according to article 69 of Regulation (EU) 2017/745 and the current national legislation regulating clinical investigation with healthcare products. Before inclusion of the patient in the trial, written informed consent will be requested. In relation to the study data, we will follow the provisions of Law 3/2018 on Personal Data Protection and guarantee of digital rights and, additionally, the General Data Protection Regulation (EU) 2016/679. Important protocol modifications (e.g. changes to eligibility criteria, outcomes, analyses) will be communicated to relevant parties after being reviewed by the ethical committee of the coordinating centre. The final trial dataset will be accessible only by the promotor, steering committee, data manager, and statistician.

### Publication of results

There is a commitment to publish the results of this study in high impact international journals, should the results be of sufficient scientific interest. No patient names will appear in any article, and no one, except for the researchers in this study and the members of the hospital ethical committees, will have access to the data, in accordance with the Law on the Protection of Data of a Personal Nature.

## Discussion

Stenosis is one of the most frequent complications in patients with CD. When an ileum resection is required, more than 50% of patients will need a repeated surgical operation after 15 years, and more than 40% will have a recurrence of obstructive symptoms after 4 years [1–3]. Thus, it is important to find alternative treatment options to surgery. EBD is the established endoscopic treatment in anastomotic stenosis. Moreover, several noncontrolled observational studies and meta-analyses have shown that EBD is safe and effective, with an overall success rate of 58–80.5% and with only 4–6% major complications [4–7]. Stents have also been used to treat stenosis in CD with reasonable efficacy and safety [15–18, 30, 31]. Our group performed the first and only clinical trial to evaluate EBD and stents (FCSEMS) for the treatment of stenosis in the CD, the ProtDilat study [19]. In this study, it was demonstrated that EBD is significantly more effective (80.5% vs 51.3%) and cheaper (EBD 1365.63 euros versus FCSEMS 1923.55 euros) than the placement of a stent, regardless of whether it is a primary or an anastomotic stricture (63% vs 71%). In contrast, the efficacy of the two treatments has been found to be similar in longer stenoses (EBD: 66.7% vs FCSEMS: 63.6%), and therefore, it appears that stents may be a suitable approach for longer stenoses in which EBD has proven to be less effective. The efficacy of FCSEMS found in our study was lower than expected, which may be partly explained by the short stricture lengths treated, with only 33% of patients with strictures longer than 4 cm. Physicians tend to restrict the indication of endoscopic treatment in clinical practice to patients with very short strictures.

Another relevant issue is the management of a stenosis that is not localized in a previous site of surgery—de novo, or primary stenosis. The ECCO guidelines consider that EBD and surgery are both suitable treatment options for terminal ileum short stenosis (< 5 cm), although there are no studies comparing the two forms of treatment [13]. Furthermore, a Spanish multicentre study led by our group, the TEDEII study, which included the largest series of CD patients treated with EBD, demonstrated that a high percentage of success was achieved with primary stenosis, similar to that seen in post-surgical stenosis [14]. In addition, the most recently published meta-analysis also corroborates the fact that EBD is equally effective, regardless of the type of stenosis [7].

In conclusion, there is currently no scientific evidence that allows us to determine the most appropriate treatment for de novo or primary stenosis (endoscopic vs surgical treatment). For this reason, we decided to carry out a clinical trial (EndoCir study) focused on the treatment of primary stenosis in CD: endoscopic treatment (first perform an EBD and if that fails then place a SEMS) vs surgical treatment. The main objective of the study will be to evaluate patients’ quality of life.

The EndoCir trial is supported by the GETECCU and FSEED (Foundation of Spanish Society of Digestive Endoscopy) and includes fifteen tertiary and secondary centres, as well as experts in the management of inflammatory bowel disease. The researchers of the Hospital Universitari Mutua Terrassa have assumed the leadership and a primary role in centralizing the decisions in case of doubts and controversies, and in limiting heterogeneity.

## Trial status

Protocol of submitted version, number and date: number 1.2; date September 2022.

Recruitment: date of first enrolment: November 28, 2022, and recruitment will be completed by November, 2024.

Revision chronology:

a-EndoCir April, 2019, original: version 1, first draft of the study protocol.

b-EndoCir September, 2022, amendment n° 1: version 1.2 - definitive.

- Minor changes: changes in statistical analysis methods and cost analysis.
- New variables added: faecal calprotectin (optional) to evaluate the clinical activity of the disease.
- Updated list of the most common pharmacological treatments in CD and Patient Reported Outcomes Measures (PROMs) added: IBD-Control questionnaire.

## Supplementary Information

**Additional file 1.** SPIRITpirit checklist.

**Additional file 2.** IBDQ-32, EQ-5D-5L questionnaires and IBD-Control (in Spanish).

## Data Availability

Minimal dataset necessary to interpret the findings available from the corresponding author on reasonable request.

## Abbreviations

AE: Adverse event
AEG: Asociación Española de Gastroenterología – Spanish Society of Gastroenterology
ASGE: American Society of Gastrointestinal Endoscopy
CD: Crohn’s disease
CDAI: Crohn’s disease activity index
CRP: C-reactive protein
CEIC: Clinical Research Ethics Committees
CRF: Case report form
EBD: Endoscopic balloon dilation
ECCO: European Crohn’s and Colitis Organization
ESR: Erythrocyte sedimentation rate
EQ-5D-5L: EuroQol 5-Dimension 5-Level
FCSEMS: Fully covered self-expandable metal stents
FSEED: Fundación de la Sociedad Española de Endoscopia Digestiva – Foundation of Spanish Society of Digestive Endoscopy
GETECCU: Spanish Working Group on Crohn’s disease and Ulcerative Colitis
IBD: Inflammatory bowel disease
IBDQ-32: 32-Item Inflammatory Bowel Disease Questionnaire
MaRIAs: Simplified magnetic resonance index of activity
MRE: Magnetic resonance enterography
PROM: Patient-reported outcome measure
REDCap: Research Electronic Data Capture
RR: Relative risk
SES-CD: Simple endoscopic score for Crohn’s disease
SR: Surgical resection
SEMS: Self-expandable metal stents
SSU: Short stay unit
