# Supplementary material for: Endoscopic treatment (endoscopic balloon dilation/self-expandable metal stent) vs surgical resection for the treatment of de novo stenosis in Crohn’s disease (ENDOCIR study): an open-label, multicentre, randomized trial
Source: Trials. 2023 Jun 27;24:432. doi: 10.1186/s13063-023-07447-1 (PMC10294410; doi:10.1186/s13063-023-07447-1)
Supplement: Supplementary file 2 — Additional file 2. IBDQ-32, EQ-5D-5L questionnaires and IBD-Control (in Spanish). [file 13063_2023_7447_MOESM2_ESM.pdf]

Anexo. Cuestionario IBDQ-32 traducido, adaptado y validado al español

MARQUE CON UN CÍRCULO LA RESPUESTA QUE CORRESPONDE MÁS EXACTAMENTE CON SU SITUACIÓN DURANTE LAS ÚLTIMAS DOS SEMANAS

1. ¿Con qué frecuencia ha ido de vientre durante las últimas dos semanas?
- ❑ 1. Más frecuentemente que nunca
- ❑ 2. Extremada frecuencia
- ❑ 3. Con mucha frecuencia
- ❑ 4. Moderado aumento de la frecuencia de defecación
- ❑ 5. Ligero aumento de la frecuencia de defecación
- ❑ 6. Aumento mínimo de la frecuencia de defecación
- ❑ 7. Normal, sin ningún aumento de la frecuencia de defecación

2. ¿Con qué frecuencia le ha causado problemas la sensación de fatiga o de cansancio y agotamiento durante las últimas dos semanas?
- ❑ 1. Siempre
- ❑ 2. Casi siempre
- ❑ 3. Bastantes veces
- ❑ 4. A veces
- ❑ 5. Pocas veces
- ❑ 6. Casi nunca
- ❑ 7. Nunca

3. ¿Con qué frecuencia se ha sentido frustrado, impaciente o inquieto a causa de su problema intestinal durante las últimas dos semanas?
- ❑ 1. Siempre
- ❑ 2. Casi siempre
- ❑ 3. Bastantes veces
- ❑ 4. A veces
- ❑ 5. Pocas veces
- ❑ 6. Casi nunca
- ❑ 7. Nunca

4. ¿Con qué frecuencia se ha visto incapacitado para ir a estudiar o al trabajo a causa de su problema intestinal durante las últimas dos semanas?
- ❑ 1. Siempre
- ❑ 2. Casi siempre
- ❑ 3. Bastantes veces
- ❑ 4. A veces
- ❑ 5. Pocas veces
- ❑ 6. Casi nunca
- ❑ 7. Nunca

5. ¿Durante cuánto tiempo en las últimas dos semanas ha tenido diarrea?
- ❑ 1. Siempre
- ❑ 2. Casi siempre
- ❑ 3. Bastantes veces
- ❑ 4. A veces
- ❑ 5. Pocas veces
- ❑ 6. Casi nunca
- ❑ 7. Nunca

6. ¿Cuánta energía ha tenido durante las últimas dos semanas?
- ❑ 1. Ninguna energía
- ❑ 2. Muy poca energía
- ❑ 3. Poca energía
- ❑ 4. Cierta energía
- ❑ 5. Bastante energía
- ❑ 6. Mucha energía
- ❑ 7. Rebosante de energía

17. En general, ¿hasta qué punto ha sido un problema tener gases durante las últimas dos semanas?
- ❑ 1. Un gran problema
- ❑ 2. Un problema importante
- ❑ 3. Bastante problemático
- ❑ 4. Algo problemático
- ❑ 5. Muy poco problemático
- ❑ 6. Casi ningún problema
- ❑ 7. Ningún problema

18. En general, ¿hasta qué punto ha sido un problema durante las últimas dos semanas el mantener o llegar al peso que a Vd. le gustaría?
- ❑ 1. Un gran problema
- ❑ 2. Un problema importante
- ❑ 3. Bastante problemático
- ❑ 4. Algo problemático
- ❑ 5. Muy poco problemático
- ❑ 6. Casi ningún problema
- ❑ 7. Ningún problema

19. Muchos pacientes con un problema intestinal tienen frecuentes preocupaciones y angustias a causa de su enfermedad. En general, ¿con qué frecuencia durante las últimas dos semanas se ha sentido preocupado o angustiado por llegar a tener cáncer, o por pensar que nunca más volvería a encontrarse bien, o por tener una recaída?
- ❑ 1. Siempre
- ❑ 2. Casi siempre
- ❑ 3. Bastantes veces
- ❑ 4. A veces
- ❑ 5. Pocas veces
- ❑ 6. Casi nunca
- ❑ 7. Nunca

20. ¿Con qué frecuencia durante las últimas dos semanas ha tenido una sensación de hinchazón abdominal?
- ❑ 1. Siempre
- ❑ 2. Casi siempre
- ❑ 3. Bastantes veces
- ❑ 4. A veces
- ❑ 5. Pocas veces
- ❑ 6. Casi nunca
- ❑ 7. Nunca

21. ¿Con qué frecuencia durante las últimas dos semanas se ha sentido relajado y libre de tensión?
- ❑ 1. Nunca
- ❑ 2. Casi nunca
- ❑ 3. Pocas veces
- ❑ 4. A veces
- ❑ 5. Bastantes veces
- ❑ 6. Casi siempre
- ❑ 7. Siempre

22. ¿Cuántas veces durante las últimas dos semanas ha tenido problemas de sangrar al ir de vientre?
- ❑ 1. Siempre
- ❑ 2. Casi siempre
- ❑ 3. Bastantes veces
- ❑ 4. A veces
- ❑ 5. Pocas veces
- ❑ 6. Casi nunca
- ❑ 7. Nunca

7. ¿Con qué frecuencia ha estado preocupado ante la posibilidad de tener que operarse por su problema intestinal durante las últimas dos semanas?
- ❑ 1. Siempre
- ❑ 2. Casi siempre
- ❑ 3. Bastantes veces
- ❑ 4. A veces
- ❑ 5. Pocas veces
- ❑ 6. Casi nunca
- ❑ 7. Nunca

8. ¿Con qué frecuencia ha tenido que aplazar o anular una cita o compromiso social a causa de su problema intestinal durante las últimas dos semanas?
- ❑ 1. Siempre
- ❑ 2. Casi siempre
- ❑ 3. Bastantes veces
- ❑ 4. A veces
- ❑ 5. Pocas veces
- ❑ 6. Casi nunca
- ❑ 7. Nunca

9. ¿Con qué frecuencia ha tenido retortijones durante las últimas dos semanas?
- ❑ 1. Siempre
- ❑ 2. Casi siempre
- ❑ 3. Bastantes veces
- ❑ 4. A veces
- ❑ 5. Pocas veces
- ❑ 6. Casi nunca
- ❑ 7. Nunca

10. ¿Con qué frecuencia ha tenido malestar general durante las últimas dos semanas?
- ❑ 1. Siempre
- ❑ 2. Casi siempre
- ❑ 3. Bastantes veces
- ❑ 4. A veces
- ❑ 5. Pocas veces
- ❑ 6. Casi nunca
- ❑ 7. Nunca

11. ¿Con que frecuencia ha estado preocupado por temor a no encontrar un lavabo cerca durante las últimas dos semanas?
- ❑ 1. Siempre
- ❑ 2. Casi siempre
- ❑ 3. Bastantes veces
- ❑ 4. A veces
- ❑ 5. Pocas veces
- ❑ 6. Casi nunca
- ❑ 7. Nunca

12. ¿Qué dificultad ha tenido, a causa de su problema intestinal, en las actividades de ocio o deportes que le hubiera gustado hacer durante las últimas dos semanas?
- ❑ 1. Muchísima dificultad; imposible hacer actividades
- ❑ 2. Mucha dificultad
- ❑ 3. Bastante dificultad
- ❑ 4. Algo de dificultad
- ❑ 5. Un poco de dificultad
- ❑ 6. Apenas ninguna dificultad
- ❑ 7. Ninguna dificultad; mi problema intestinal no ha limitado mis actividades de ocio ni deportivas

23. ¿Con qué frecuencia durante las últimas dos semanas se ha sentido avergonzado en público por olores desagradables o ruidos causados por su problema intestinal?
- ❑ 1. Siempre
- ❑ 2. Casi siempre
- ❑ 3. Bastantes veces
- ❑ 4. A veces
- ❑ 5. Pocas veces
- ❑ 6. Casi nunca
- ❑ 7. Nunca

24. ¿Con qué frecuencia durante las últimas dos semanas ha tenido ganas de ir al lavabo sin realmente hacer de vientre?
- ❑ 1. Siempre
- ❑ 2. Casi siempre
- ❑ 3. Bastantes veces
- ❑ 4. A veces
- ❑ 5. Pocas veces
- ❑ 6. Casi nunca
- ❑ 7. Nunca

25. ¿Con qué frecuencia se ha sentido deprimido, lloroso o desanimado a causa de su problema intestinal durante las últimas dos semanas?
- ❑ 1. Siempre
- ❑ 2. Casi siempre
- ❑ 3. Bastantes veces
- ❑ 4. A veces
- ❑ 5. Pocas veces
- ❑ 6. Casi nunca
- ❑ 7. Nunca

26. ¿Con qué frecuencia durante las últimas dos semanas ha manchado accidentalmente su ropa interior?
- ❑ 1. Siempre
- ❑ 2. Casi siempre
- ❑ 3. Bastantes veces
- ❑ 4. A veces
- ❑ 5. Pocas veces
- ❑ 6. Casi nunca
- ❑ 7. Nunca

27. ¿Con qué frecuencia durante las últimas dos semanas se ha sentido enfadado a causa de su problema intestinal?
- ❑ 1. Siempre
- ❑ 2. Casi siempre
- ❑ 3. Bastantes veces
- ❑ 4. A veces
- ❑ 5. Pocas veces
- ❑ 6. Casi nunca
- ❑ 7. Nunca

28. En general, durante las últimas dos semanas, ¿hasta qué punto su problema intestinal ha sido un problema para sus relaciones sexuales?
- ❑ 1. Un gran problema
- ❑ 2. Un problema importante
- ❑ 3. Bastante problemático
- ❑ 4. Algo problemático
- ❑ 5. Muy poco problemático
- ❑ 6. Casi ningún problema
- ❑ 7. Ningún problema

13. ¿Con qué frecuencia ha tenido dolor abdominal durante las últimas dos semanas?
- ❑ 1. Siempre
- ❑ 2. Casi siempre
- ❑ 3. Bastantes veces
- ❑ 4. A veces
- ❑ 5. Pocas veces
- ❑ 6. Casi nunca
- ❑ 7. Nunca

14. ¿Con qué frecuencia ha tenido problemas porque se ha despertado por la noche durante las últimas dos semanas?
- ❑ 1. Siempre
- ❑ 2. Casi siempre
- ❑ 3. Bastantes veces
- ❑ 4. A veces
- ❑ 5. Pocas veces
- ❑ 6. Casi nunca
- ❑ 7. Nunca

15. ¿Con qué frecuencia se ha sentido deprimido o desanimado a causa de su problema intestinal durante las últimas dos semanas?
- ❑ 1. Siempre
- ❑ 2. Casi siempre
- ❑ 3. Bastantes veces
- ❑ 4. A veces
- ❑ 5. Pocas veces
- ❑ 6. Casi nunca
- ❑ 7. Nunca

16. ¿Con qué frecuencia durante las últimas dos semanas ha tenido que dejar de asistir a actos sociales porque no había un lavabo cerca?
- ❑ 1. Siempre
- ❑ 2. Casi siempre
- ❑ 3. Bastantes veces
- ❑ 4. A veces
- ❑ 5. Pocas veces
- ❑ 6. Casi nunca
- ❑ 7. Nunca

29. ¿Con qué frecuencia ha tenido náuseas o ganas de vomitar durante las últimas dos semanas?
- ❑ 1. Siempre
- ❑ 2. Casi siempre
- ❑ 3. Bastantes veces
- ❑ 4. A veces
- ❑ 5. Pocas veces
- ❑ 6. Casi nunca
- ❑ 7. Nunca

30. ¿Con qué frecuencia se ha sentido de mal humor durante las últimas dos semanas?
- ❑ 1. Siempre
- ❑ 2. Casi siempre
- ❑ 3. Bastantes veces
- ❑ 4. A veces
- ❑ 5. Pocas veces
- ❑ 6. Casi nunca
- ❑ 7. Nunca

31. ¿Con qué frecuencia durante las últimas dos semanas se ha sentido incomprendido por los demás?
- ❑ 1. Siempre
- ❑ 2. Casi siempre
- ❑ 3. Bastantes veces
- ❑ 4. A veces
- ❑ 5. Pocas veces
- ❑ 6. Casi nunca
- ❑ 7. Nunca

32. ¿Hasta qué punto ha estado satisfecho, contento o feliz con su vida personal durante las últimas dos semanas?
- ❑ 1. Muy insatisfecho, infeliz
- ❑ 2. Bastante insatisfecho, infeliz
- ❑ 3. Algo insatisfecho, descontento
- ❑ 4. Algo satisfecho, contento
- ❑ 5. Bastante satisfecho, contento
- ❑ 6. Muy satisfecho, feliz
- ❑ 7. Extremadamente satisfecho, no podría ser más feliz

Cuestionario de Salud

Versión en español para España

(Spanish version for Spain)

Spain (Spanish) © 2009 EuroQol Group EQ-5D™ is a trade mark of the EuroQol Group

Debajo de cada enunciado, marque UNA casilla, la que mejor describe su salud HOY.

MOVILIDAD

No tengo problemas para caminar	❑
Tengo problemas leves para caminar	❑
Tengo problemas moderados para caminar	❑
Tengo problemas graves para caminar	❑
No puedo caminar	❑

AUTO-CUIDADO

No tengo problemas para lavarme o vestirme	❑
Tengo problemas leves para lavarme o vestirme	❑
Tengo problemas moderados para lavarme o vestirme	❑
Tengo problemas graves para lavarme o vestirme	❑
No puedo lavarme o vestirme	❑

ACTIVIDADES COTIDIANAS *(Ej.: trabajar, estudiar, hacer las tareas domésticas, actividades familiares o actividades durante el tiempo libre)*

No tengo problemas para realizar mis actividades cotidianas	❑
Tengo problemas leves para realizar mis actividades cotidianas	❑
Tengo problemas moderados para realizar mis actividades cotidianas	❑
Tengo problemas graves para realizar mis actividades cotidianas	❑
No puedo realizar mis actividades cotidianas	❑

DOLOR / MALESTAR

No tengo dolor ni malestar	❑
Tengo dolor o malestar leve	❑
Tengo dolor o malestar moderado	❑
Tengo dolor o malestar fuerte	❑
Tengo dolor o malestar extremo	❑

ANSIEDAD / DEPRESIÓN

No estoy ansioso ni deprimido	❑
Estoy levemente ansioso o deprimido	❑
Estoy moderadamente ansioso o deprimido	❑
Estoy muy ansioso o deprimido	❑
Estoy extremadamente ansioso o deprimido	❑

Spain (Spanish) © 2009 EuroQol Group EQ-5D™ is a trade mark of the EuroQol Group

- Nos gustaría conocer lo buena o mala que es su salud HOY.
- La escala está numerada del 0 al 100.
- 100 representa la mejor salud que usted se pueda imaginar.
  0 representa la peor salud que usted se pueda imaginar.
- Marque con una X en la escala para indicar cuál es su estado de salud HOY.
- Ahora, en la casilla que encontrará a continuación escriba el número que ha marcado en la escala.

SU SALUD HOY = [ ]

La mejor salud que usted se pueda imaginar

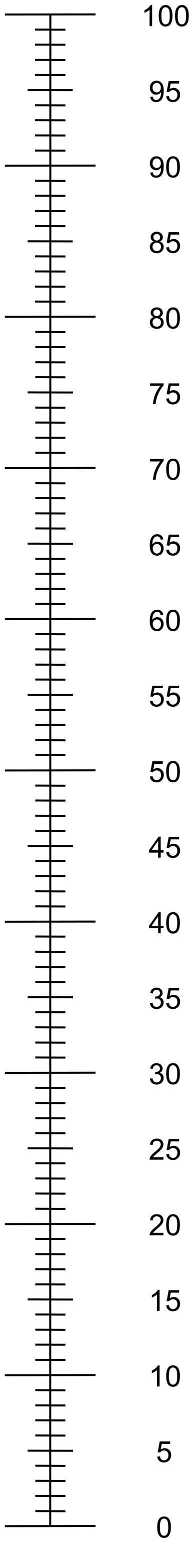

La peor salud que usted se pueda imaginar

Spain (Spanish) © 2009 EuroQol Group EQ-5D™ is a trade mark of the EuroQol Group

EII-Control

Cuestionario para el control de la enfermedad inflamatoria intestinal

1. ¿Usted considera que…

	Sí	No	No estoy seguro
**a. su EII ha estado bien controlada en las últimas dos semanas?**	☐	☐	☐
**b. su tratamiento actual es útil para controlar su EII?**	☐	☐	☐

Si no está tomando ningún tratamiento, marque esta casilla ☐

	Mejor	Sin cambios	Peor
**2. Durante las últimas dos semanas, sus síntomas intestinales, ¿han mejorado, han empeorado o no han sufrido ningún cambio?**	☐	☐	☐

3. En las últimas dos semanas, usted….

	Sí	No	No estoy seguro
**a. ha dejado de hacer alguna actividad planeada por culpa de su EII?** ***(por ej. acudir al trabajo, asistir a sus clases, actos sociales…)***	☐	☐	☐
**b. se ha despertado por la noche por sus síntomas de EII?**	☐	☐	☐
**c. ha sufrido un dolor o malestar significativo?**	☐	☐	☐
**d. se ha sentido a menudo fatigado o sin fuerzas?** ***(por a menudo, nos referimos a más de la mitad del tiempo)***	☐	☐	☐
**e. se ha sentido ansioso o deprimido como consecuencia de su EII?**	☐	☐	☐
**f. ha creído que necesitaba un cambio en su tratamiento?**	☐	☐	☐

4. En su próxima visita al médico, ¿qué temas le gustaría tratar?

	Sí	No	No estoy seguro
**a. tipos de medicamentos alternativos para controlar la EII**	☐	☐	☐
**b. formas de ajustar su propio tratamiento**	☐	☐	☐
**c. efectos secundarios asociados con el uso de sus medicamentos**	☐	☐	☐
**d. nuevos síntomas que han aparecido desde su última visita**	☐	☐	☐

5. Finalmente, ¿cómo valoraría usted el control global de su EII durante las últimas dos semanas?

Utilice la siguiente escala de 0 (peor control posible) a 100 (mejor control posible) señalando con una línea vertical en la puntuación que corresponda

Peor control posible | 0 — 10 — 20 — 30 — 40 — 50 — 60 — 70 — 80 — 90 — 100 | Mejor control posible
